# An artificial intelligence-based approach for identifying rare disease patients using retrospective electronic health records applied for Pompe disease

**DOI:** 10.3389/fneur.2023.1108222

**Published:** 2023-04-21

**Authors:** Simon Lin, Jama Nateqi, Rafael Weingartner-Ortner, Stefanie Gruarin, Hannes Marling, Vinzenz Pilgram, Florian B. Lagler, Elmar Aigner, Alistair G. Martin

**Affiliations:** ^1^Science Department, Symptoma GmbH, Vienna, Austria; ^2^Department of Internal Medicine, Paracelsus Medical University, Salzburg, Austria; ^3^Science Department, Symptoma GmbH, Salzburg, Austria; ^4^Medical and Information Technology - MIT, University Hospital Salzburg (SALK), Salzburg, Austria; ^5^Department of Pediatrics and Institute for Inherited Metabolic Diseases, Paracelsus Medical University, Salzburg, Austria

**Keywords:** electronic health records (EHR), artificial intelligence (AI), Pompe disease (glycogen storage disease type II), rare disease (RD), orphan disease, retrospective screening

## Abstract

**Objective:**

We retrospectively screened 350,116 electronic health records (EHRs) to identify suspected patients for Pompe disease. Using these suspected patients, we then describe their phenotypical characteristics and estimate the prevalence in the respective population covered by the EHRs.

**Methods:**

We applied Symptoma's Artificial Intelligence-based approach for identifying rare disease patients to retrospective anonymized EHRs provided by the “University Hospital Salzburg” clinic group. Within 1 month, the AI screened 350,116 EHRs reaching back 15 years from five hospitals, and 104 patients were flagged as probable for Pompe disease. Flagged patients were manually reviewed and assessed by generalist and specialist physicians for their likelihood for Pompe disease, from which the performance of the algorithms was evaluated.

**Results:**

Of the 104 patients flagged by the algorithms, generalist physicians found five “diagnosed,” 10 “suspected,” and seven patients with “reduced suspicion.” After feedback from Pompe disease specialist physicians, 19 patients remained clinically plausible for Pompe disease, resulting in a specificity of 18.27% for the AI. Estimating from the remaining plausible patients, the prevalence of Pompe disease for the greater Salzburg region [incl. Bavaria (Germany), Styria (Austria), and Upper Austria (Austria)] was one in every 18,427 people. Phenotypes for patient cohorts with an approximated onset of symptoms above or below 1 year of age were established, which correspond to infantile-onset Pompe disease (IOPD) and late-onset Pompe disease (LOPD), respectively.

**Conclusion:**

Our study shows the feasibility of Symptoma's AI-based approach for identifying rare disease patients using retrospective EHRs. Via the algorithm's screening of an entire EHR population, a physician had only to manually review 5.47 patients on average to find one suspected candidate. This efficiency is crucial as Pompe disease, while rare, is a progressively debilitating but treatable neuromuscular disease. As such, we demonstrated both the efficiency of the approach and the potential of a scalable solution to the systematic identification of rare disease patients. Thus, similar implementation of this methodology should be encouraged to improve care for all rare disease patients.

## 1. Introduction

The main challenges for rare diseases (RD) are related to diagnosis because, by definition, they are unknown to patients and physicians because of their rarity (1, 2), are characterized by a broad diversity of syndromic disorders, where symptoms in isolation, can be mistaken for more common diseases, also leading to “premature closure” of the diagnostic journey (2), and prolonged diagnostic journey consulting multiple physicians and undergoing numerous examinations and treatments, impairing a conclusive review of the overtime gathered retrospective documentation. Rare diseases are defined as those with a prevalence of fewer than 200,000 people each year in the US and fewer than one per 2,000 people in the European Union (3, 4). Under these definitions, around 7,000 different conditions qualify as rare diseases, yet there are only ~20,000 overall known diseases (5). This highlights the scale of the problem of misdiagnosis in rare diseases. Above mentioned reasons altogether lead to a lengthy and burdensome path to diagnosis that can take, on average, 96 months (8 years) and even 236 months (28 years) for a quarter of patients (6). Within this manuscript, we highlight the rare disease Pompe disease. On average, Pompe disease patients wait for 2.5 months (infantile-onset) and up to 144 months (late-onset) for the right diagnosis (7, 8). Yet, this only applies to the patients who eventually are diagnosed correctly. Several studies focusing on patients suffering from myopathies of unknown etiology have shown missed diagnosis of Pompe disease to be a significant problem, highlighting the number of unknown cases where the right diagnosis is never found (9–13).

The prevalence of Pompe disease varies significantly based upon the estimation method (11, 14–17). For example, estimations based on genetic databases give a prevalence of one in every 23,232 people (1:23,232) globally (15). Whereas survey-based investigations, where clinical centers treating Pompe disease patients were contacted, calculated prevalence rates of 1:350,914 and 1:283,000, respectively (16, 17). Similar variation in prevalence is seen geographically. The estimated prevalence in Austria from genetic newborn screening results is 1:8684, whereas a broader analysis of global genetic databases suggests a prevalence of 1:13,756 for (non-Finnish) Europeans (14, 15). The highest prevalence was observed in the East Asian population (1:12,125) and the lowest in the Finnish (1:1,056,444) (15). A relative difference in the incidence of 98.85%. Such variation again highlights the limitations and challenges to assessing the prevalence of rare diseases, but Pompe disease in particular.

The typical phenotype presents itself in two types: infantile-onset Pompe disease (IOPD) and late-onset Pompe disease (LOPD), which are generally well-described (18). Within IOPD, the disease manifestation strongly correlates with the patient's genotype, specifically the level of acid α-glucosidase (GAA) activity (19). In contrast, the variable progression in LOPD is influenced by yet unknown factors (18). This genetic variance also poses challenges to the accuracy of genetic newborn screening programs. Such programs have been installed in certain regions, but are costly ($408,000/Quality of life years [QALY]) (20) and still produce high false positive (i.e., Pseudo Deficiency, Carrier, No Disorder) rates ranging from 92.52 to 79.55% (21–23). Diagnosis, and a better understanding of the mechanics of RDs, like Pompe disease, are impeded by the rare disease conundrum, where the arduous diagnosis of a RD hinders the generation of knowledge on said RDs, thereby enabling diagnosis in the first place (24). The biggest challenge is identifying suspicious patients and then routing them into the correct clinical lane for further diagnostic workup, especially in rare disease competence centers (1, 7, 25). Several studies have already shown that digital tools have the potential to support the early diagnosis of rare disease patients (26).

Despite these promising results in the published literature, many different technical, economic and political barriers cause a reduction in the uptake of medical innovations. As such, few solutions have been validated outside the academic sphere. The main facilitating factors, namely ease of use, integration into care, and user-friendliness, mentioned in the literature all revolve around the fact that the solution must not disrupt the existing processes (27, 28). Thus, building a solution which exploits existing resources and integrates into existing infrastructures and processes distinctly increases the chances of a successful uptake.

Electronic Health Records (EHRs) refer to the comprehensive collection of healthcare data for all patients. As such, EHRs represent an existing rich resource of retrospective data already present within most institutions and downstream of existing protocols. Given these features, it is no surprise that solutions based on EHRs have been widely suggested. However, unlocking the potential of data harbored in EHRs is non-trivial. The data is highly heterogenous and often incomplete, making it troublesome for traditional automated solutions. To our knowledge, no solution has reportedly achieved real-world impact so far using retrospective EHRs (26, 29–31).

Making this highly heterogenous and flawed data available for AI ingestion requires several cutting-edge technologies and is a highly active research topic (32, 33). For example, the n2c2 Clinical Challenges, a periodic release of annotated de-identified clinical notes, enables hundreds of research articles detailing how best to extract information from unstructured medical data (34). Once this data has been prepared, automated screening of EHRs can serve as a highly sensitive first step in the screening funnel for rare diseases. Automated screening has the capacity to enable large-scale rare disease patient screening, while reducing efforts of accurate patient selection, without disrupting existing workflows, therefore, increasing cost-effectiveness and ultimately the discovery rates of rare disease patients.

In this manuscript, we describe the outcomes of an automated artificial intelligence (AI)-based methodology to identify Pompe disease patients based on their existing retrospective EHRs. We present the results and compare the efficiency rates of our methodology with other comparable screening projects. Further, we discuss the phenotypical findings of identified suspected patients.

## 2. Materials and methods

### 2.1. Artificial intelligence

In our retrospective data analysis study, a proprietary AI developed by Symptoma^1^ was applied to retrospective anonymized EHRs. Symptoma designed the AI to identify patients who are likely to suffer from a specified rare disease. The performance of Symptoma's technologies has been demonstrated in previous studies (35–37). Within this study, the target was Pompe disease. A patient suggested by the AI is called “flagged.” The data features leading to a classification as “flagged” can be divided into three non-mutually exclusive general groups: clinical presentation, patient profile, and hidden disease patterns. Clinical presentation includes a symptomatic presentation and diagnostic test results (e.g., laboratory tests and imaging). Patient profile refers to age, sex, and family history. Hidden disease patterns encompass features which are not traditionally clinically relevant and are highlighted by the AI. For example, the sequence of departments visited by a patient. The performance of the AI was benchmarked *in-silico* for Pompe disease [MRR = 0.95, 95% CI (0.884–1.0); ROC-AUC = 0.987, 95% CI (0.962–1.0); F1 score (considering top 10 results) = 0.983, 95% CI (0.947–1.0)]. More detailed information on this analysis can be found in Supplementary material S1.

### 2.2. Evaluation

When the AI determines that enough evidence is present for a given patient to be suspected of Pompe disease, it flags them for further review. To assess the quality of the “Flagged” patients, their respective anonymized EHRs are first reviewed by generalist physicians (GP). Those deemed valid candidates are then presented to specialist physicians (SP) for Pompe disease. For this study, the SPs were a pediatrician and an internal medicine physician both specialized in rare metabolic diseases. The GP allocated the labels “Diagnosed,” “Suspected,” “Reduced Suspicion,” and “Rejected” (definitions in Table 1), while the SP assigned the labels “Definite,” “Probable,” “Possible,” “Inconclusive,” and “Unlikely” (definitions in Table 2). Patients labeled either “Rejected” by the GP or “Unlikely” by the SP were considered as “Negative” for further analysis. All others were considered “Positive.” Historically diagnosed patients were identified using ICD codes and disease name and were considered must-not-miss patients.

**Table 1:** Classifications by general physicians.

**Label**	**Explanation**
Diagnosed	Medical review found a documented diagnosis for the target disease
Suspected	Medical review found supporting clinical evidence for the target disease
Reduced suspicion	Medical review found conflicting clinical evidence for the target disease
Rejected	This patient will be rejected due to the medical review result overriding the AI result

**Table 2:** Classification after specialist feedback.

**Label**	**Explanation**
Definite	The targeted disease is the top differential diagnosis for the given case
Probable	The targeted disease is in the top 10 of differential diagnoses for the given case
Possible	The targeted disease cannot be rejected as a differential diagnosis for the given case
Inconclusive	There is a lack of apparent evidence speaking for or against the targeted disease as differential diagnosis for the given case
Unlikely	The targeted disease is not among the differential diagnoses for the given case

In addition to the “Flagged” cohort, a “Background” cohort was generated from the remaining patients. For each flagged patient, a random patient from those remaining was selected. Selection was biased such that those paired had similar ages and quantity of documentation.

### 2.3. Data preparation

EHR data was provided by the “University Hospital Salzburg” (Landeskliniken Salzburg, referred to as SALK^2^) clinic group with whom a data permit had been granted. Within this study, SALK prepared a total of 350,116 EHRs. Each EHR contains an array of document types (specified in Table 3) related to an individual patient. All data was anonymized by the IT department of the clinic group prior to analysis. The analysis itself was performed using Symptoma's proprietary AI. For the evaluation of patient characteristics, data was extracted from free-text documentation using Symptoma's proprietary data processing tools. To account for historical Pompe disease patients, the respective dataset was blinded by removing information directly suggesting a Pompe disease diagnosis (i.e., disease name, GAA deficiency, enzyme replacement therapy).

**Table 3:** Available document types.

1. Entries on allergies
2. Anamnesis/patient history
3. Discharge letter
4. Specialist findings
5. Entries on departments visited and case ID
6. Doctor's notes
7. ICD—codes
8. Procedure codes
9. Vitals and other measurements
10. Laboratory result values
11. List of medications
12. Surgical reports
13. Administrative patient profile
14. Nurses' notes
15. Radiology reports
16. Entries on social habits/risk factors
17. Wound reports

### 2.4. Statistical analysis

The number of patients needed to screen for one patient to be a clinically plausible Pompe disease patient, that meaning not rejected under medical review (“Positive” label), was calculated as a binomial confidence interval using the Wilson Score Interval. The Wilson score interval is appropriate due to the infrequency of patients meaning the probability that a random patient suffers from Pompe disease is near zero. To identify any separation between the “Positive,” “Negative,” and “Background” cohorts, we performed a principal component analysis (PCA). To analyze the characteristics of these clinically plausible patients, we subset to those features associated with at least four patients in our flagged patient cohort. We calculated the association of patient characteristics to Pompe disease via the Fisher exact test, reporting the odds ratios within the text. We test both “Positive” against “Negative and “Positive” vs. “Background.” Lastly, we investigated the association between patient characteristics for the 10 most differentiating features according to the “Positive” vs. “Background” analysis above. The co-occurrence, the percentage of patients with both symptoms, is reported alongside the odds ratio. To account for multiple testing, *p*-values were corrected via the Holm-Sidak method throughout our manuscript.

## 3. Results

### 3.1. Number needed to screen

**Figure 1:**
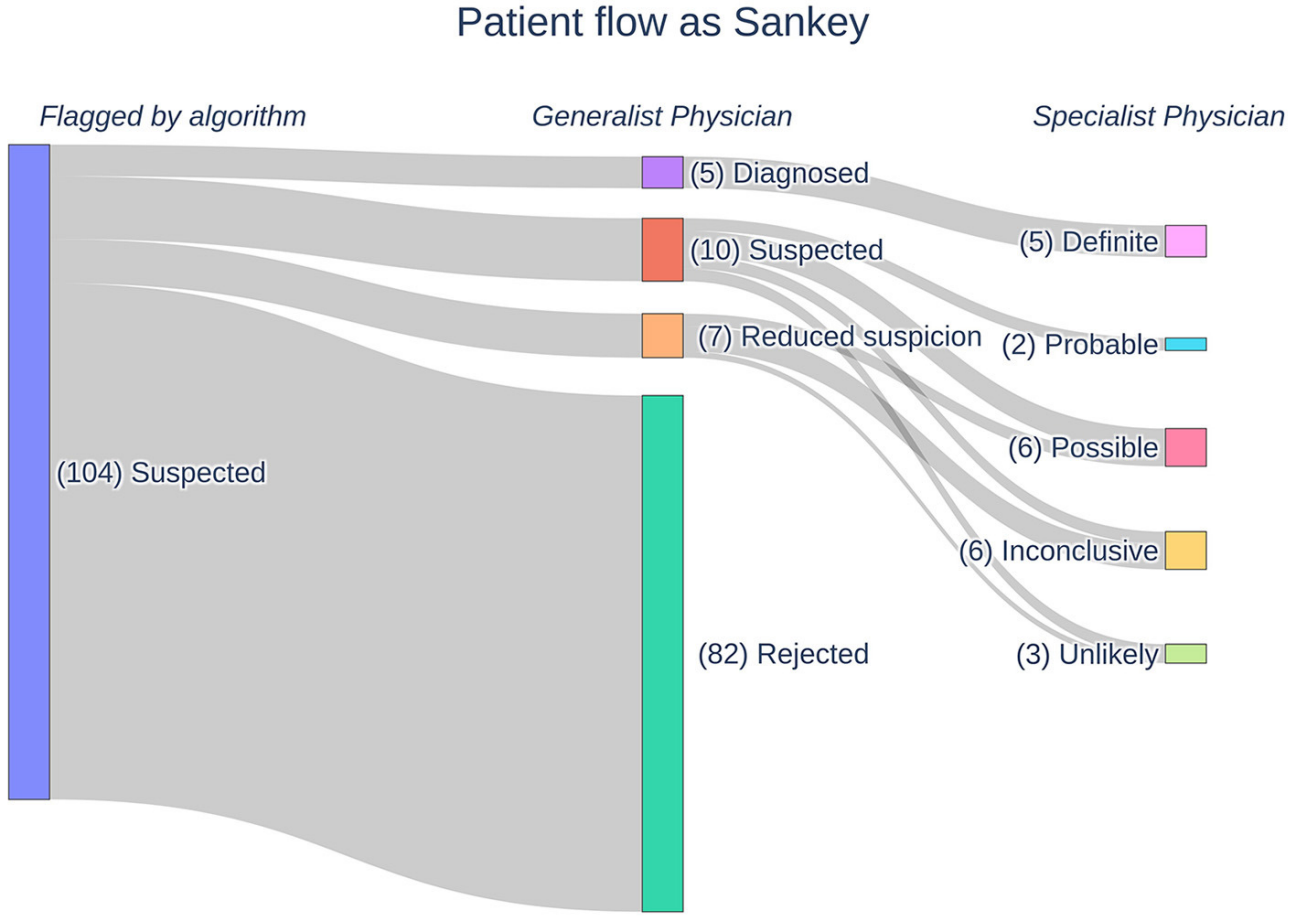
A Sankey diagram outlining the flow of patients in our Pompe disease process funnel. Patients are flagged by our AI following which they undergo manual medical reviews by generalist and specialist physicians. The physicians assign labels to the patients according to their respective likelihoods for Pompe disease.

The AI identified (flagged) 104 suspicious patients out of the pool of 350,116 patients based on their retrospective EHRs. Therefore, the AI found one suspicious patient for every 3,366.5 (95% CI: 2,778.8–4,078.6) patients screened. These patients underwent medical review by the GPs, which reduced the number of suspected patients to 22. If one considers the removed patients as false positives, the AI has a specificity of 21.15%. The GPs further divided the patients into five “diagnosed,” 10 “suspected,” and seven “reduced suspicion” patients. In the consecutive feedback round, the SPs assigned the patients into five “definite,” two “probable,” six “possible,” six “inconclusive,” and three “unlikely” patients. In Figure 1, a Sankey diagram describes the flow of patients in the process funnel alongside the labeling by the consecutive reviews. Adding those considered “unlikely” by the SPs to the other false positive patients, namely those flagged by the AI but rejected by the GPs, gives a specificity of 18.27% for the AI. The prevalence of Pompe disease patients within SALK based upon these results is 1:18,427.16 (95% CI: 11,797.67–28,782.29).

### 3.2. Patient characteristics

**Figure 2:**
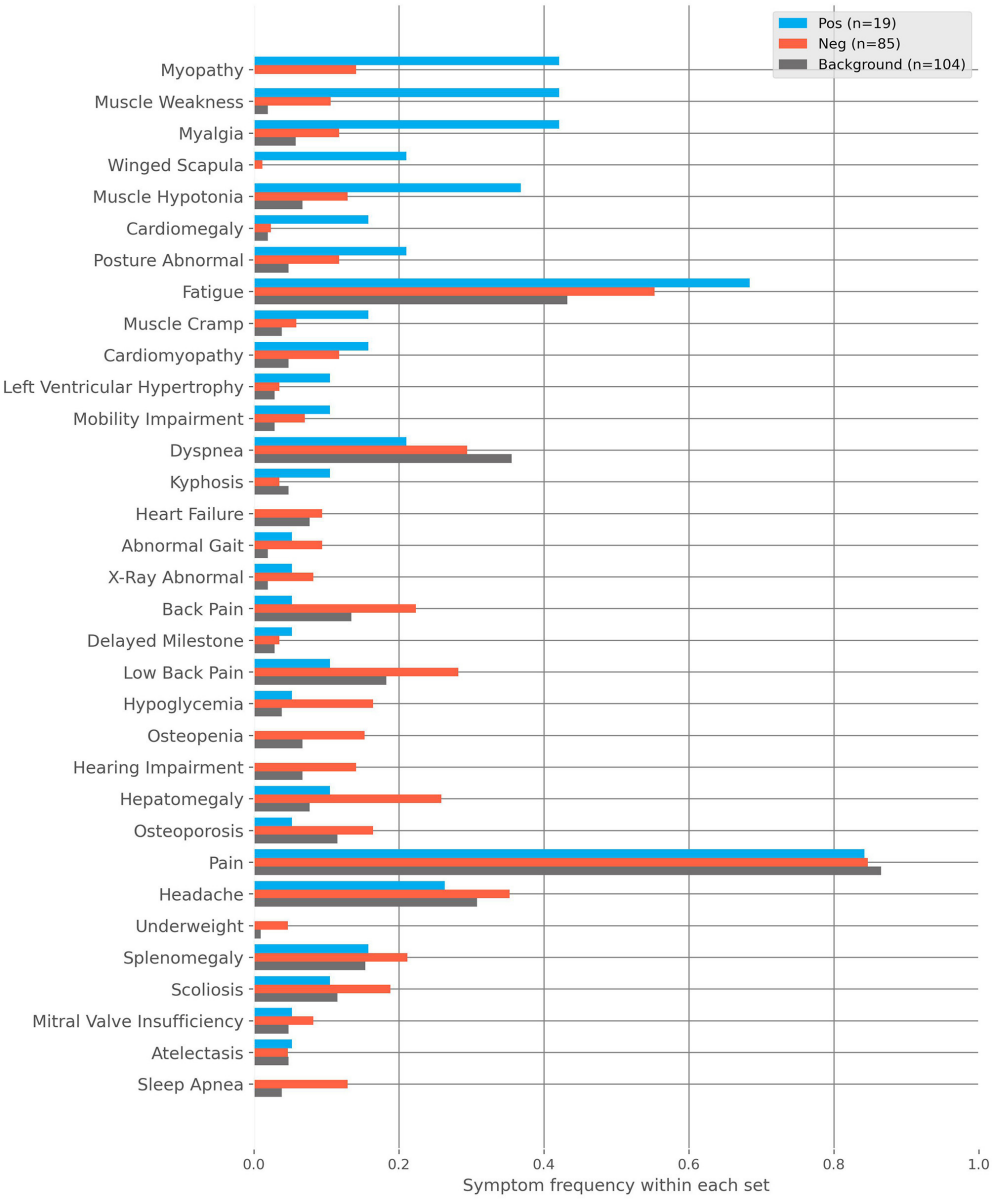
The frequencies of characteristics found within the positive, negative and background cohorts. The positive cohort are those flagged by the AI and then deemed clinically plausible for Pompe disease after medical review. The negative cohort are those flagged by the AI and then rejected with regard to Pompe disease after medical review. The background cohort is sampled from the remaining non-flagged patients.

For further analysis, we divided flagged patients into two groups, positive and negative. Patients rejected during GP review or received the label “unlikely” from SPs later were regarded as negative (*n* = 85). All remaining patients were regarded as positive (*n* = 19). Figure 2 shows the frequencies of characteristics found in these groups as well as those found within our randomly sampled background cohort. The most prevalent patient characteristics within the flagged cohort were pain, fatigue, headache, hepatomegaly, and dyspnea (*n* = 83, *n* = 50, *n* = 32, *n* = 24, and *n* = 23, respectively). The top five most differentiating characteristics between the positive and negative cohorts are:

- Muscle Weakness: OR = 6.14, *p*-value (corrected) = 0.08.
- Scapula Alata: OR = 22.4, *p*-value (corrected) = 0.112.
- Myalgia: OR = 5.45, *p*-value (corrected) = 0.122.
- Myopathy: OR = 4.42, *p*-value (corrected) = 0.251.
- Muscle Hypotonia: OR = 3.92, *p*-value (corrected) = 0.450.

Similarly, the top five most differentiating characteristics between the positive and background cohorts are:

- Myopathy: OR = Inf, *p*-value (corrected) = 2.4E-6.
- Muscle Weakness: OR = 37.09, *p*-value (corrected) = 8.84E-5.
- Myalgia: OR = 11.88, *p*-value (corrected) = 0.004.
- Scapula Alata: OR = Inf, *p*-value (corrected) = 0.013.
- Muscle Hypotonia: OR = 8.08, *p*-value (corrected) = 0.035.

Please note that the infinite odds ratio (OR) associated with Myopathy and Scapula Alata is due to those characteristics only being found in the positive cohort.

### 3.3. Principal component analysis

**Figure 3:**
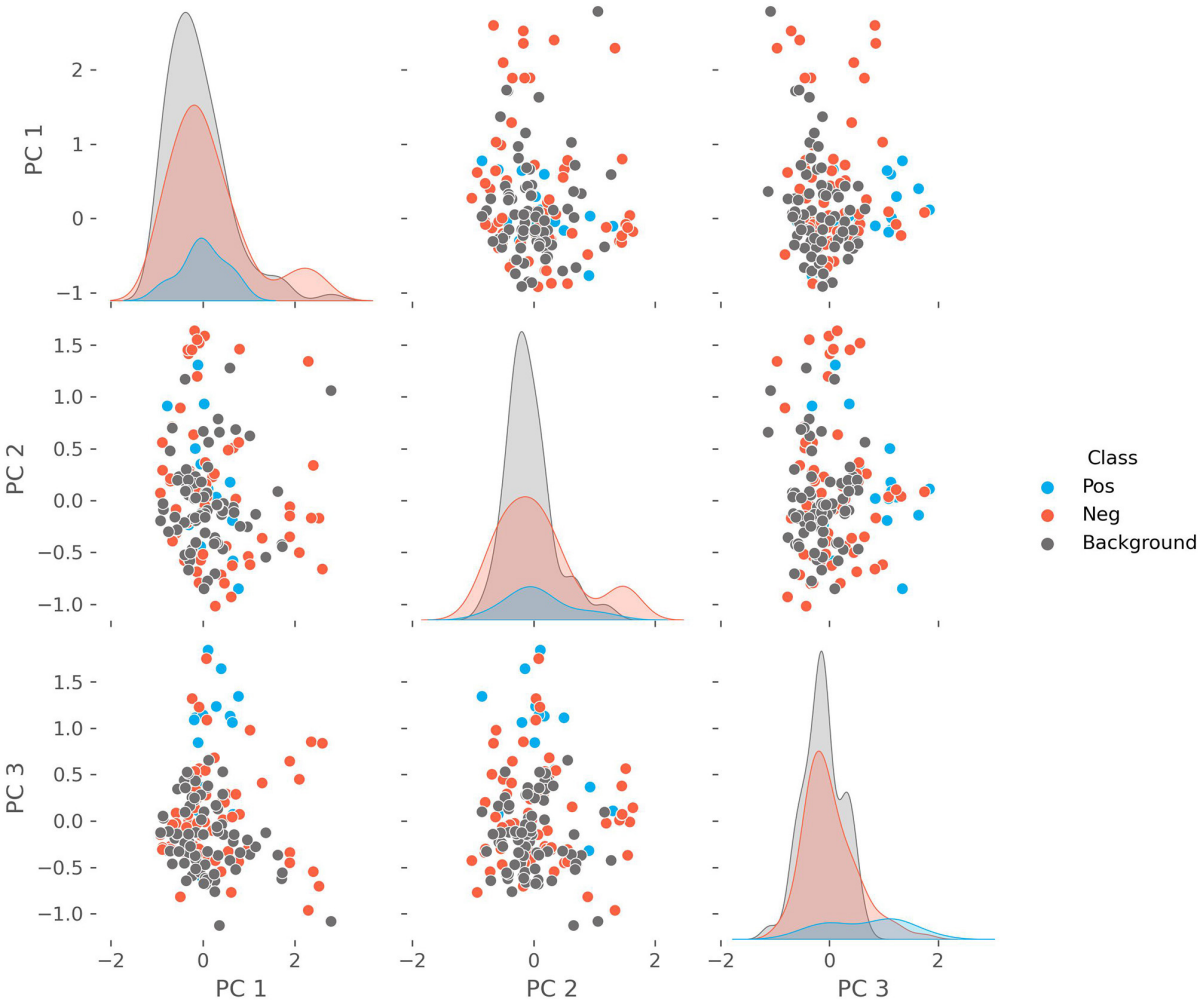
The pairwise plots of the first three principal components alongside their respective densities of a principal component analysis performed on the combined positive, negative, and background cohorts. The features with the largest contributions to the variance of the first principal component (PC1) are: “Fatigue, Headache, Lower Back Pain”; to the PC2: “Splenomegaly, Hepatomegaly, Fatigue”; and to the PC3: “Fatigue, Myalgia, Dyspnea.”

We performed a PCA on the patient characteristics to reduce the dimensionality, explore the separation with the background population, and identify potential patient clusters. Clustered patients within the positive cohort may represent different phenotypic presentations. The outcome is shown in Figure 3, where we show the first three principal components and the density with respect to each cohort (positive, negative, and background). The explained variance for each of the first three components is 0.167, 0.085, and 0.074, respectively. The features with the largest contributions to the first principal component (PC1) are: “Fatigue, Headache, Lower Back Pain”; to the PC2: “Splenomegaly, Hepatomegaly, Fatigue”; and to the PC3: “Fatigue, Myalgia, Dyspnea.” We find that PC3 drives a weak separation of the positive and other groups.

### 3.4. Co-occurrence analysis

**Figure 4:**
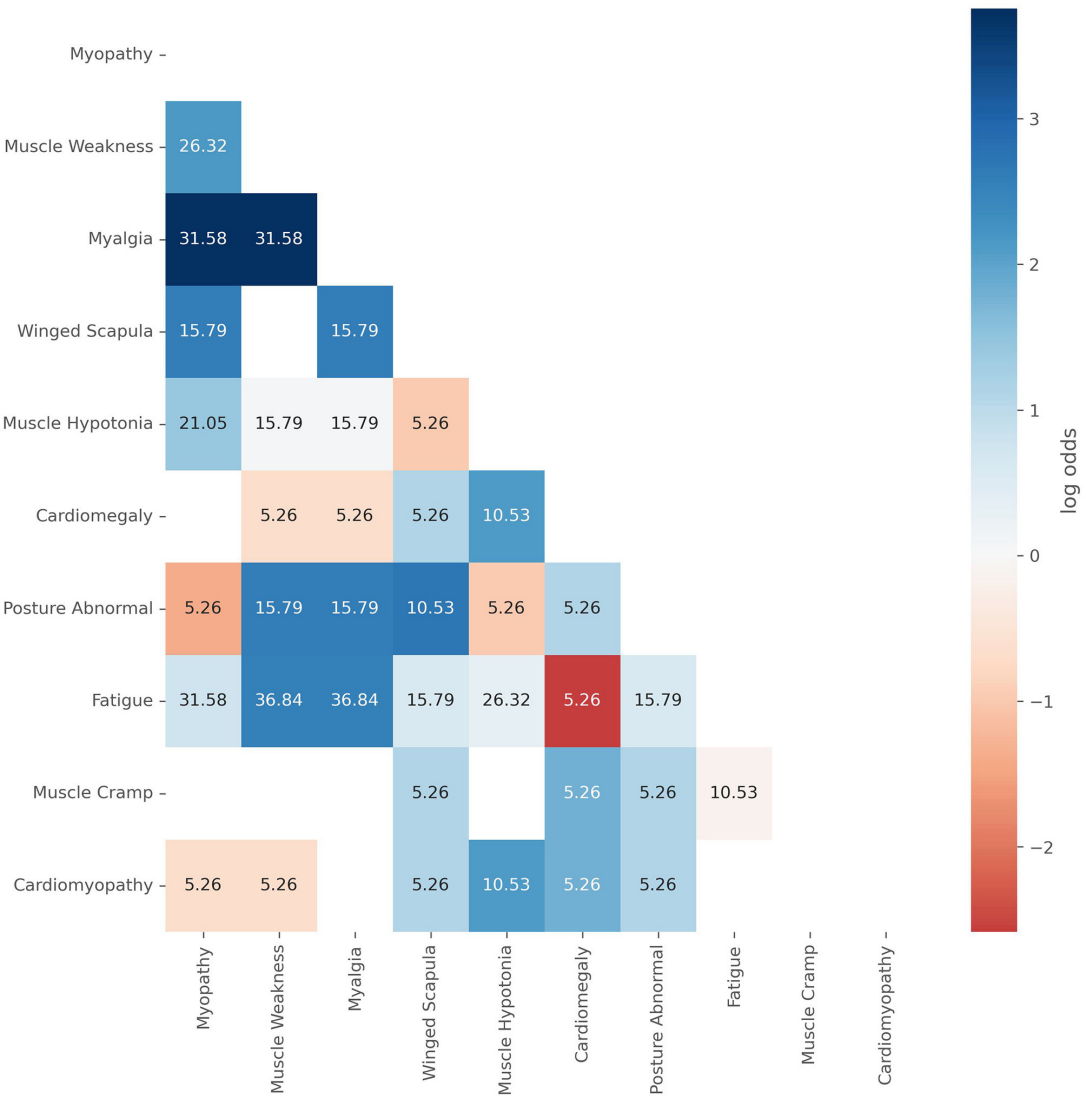
The co-occurrence, given as the text annotation, and the log odds ratio (LOR) of characteristics found within the positive cohort as color code. The co-occurrence is the percentage of the cohort with both of a given characteristic while the LOR indicates if that characteristic pairing appears more or less than expected by chance, given their independent rates of occurrence. A LOR above zero indicates enrichment for a given pairing, while a value lower than zero indicates the pairing occurs less than expected. All elements left blank have an infinite LOR as the characteristics always occur together.

The heatmap (Figure 4) shows the co-occurrence (CO) and odds ratios of patient characteristics pairings within the positive cohort. The annotation for each element is the co-occurrence, the percentage of patients in which both characteristics are present. The color encodes the respective logarithmic odds ratios (LOR), indicating which characteristic pairs appear more or less than expected by chance, given their independent rates of occurrence. A LOR above zero indicates enrichment for a given pairing, while a value lower than zero indicates the pairing occurs less than expected. All elements left blank have an infinite LOR as the characteristics always occur together. The characteristic pairs which always occur together (LOR = Inf) are:

- Scapula Alata and Muscle Weakness [CO = 21.1%, *p*-value (corrected) = 0.55],
- Cardiomegaly and Myopathy [CO = 15.8%, *p*-value (corrected) = 0.91],
- Muscle Cramps and Myopathy [CO = 15.8%, *p*-value (corrected) = 0.91],
- Muscle Cramps and Muscle Weakness [CO = 15.8%, *p*-value (corrected) = 0.91],
- Muscle Cramps and Myalgia [CO = 15.8%, *p*-value (corrected) = 0.91],
- Muscle Cramps and Muscle Hypotonia [CO = 15.8%, *p-*value (corrected) = 0.91],
- Cardiomyopathy and Fatigue [CO = 15.8%, *p*-value (corrected) = 1.00].

### 3.5. Phenotypes

In a sub-analysis, we have looked at the phenotypes in the age groups related to IOPD and LOPD, which correspond to symptom onset before or post 1 year of age, respectively (19). Due to the inconsistency of precise ages of onset in some cases, which is a commonly encountered problem in rare disease patients (19), we approximated the age of onset as the age at the first admission.

**Figure 5:**
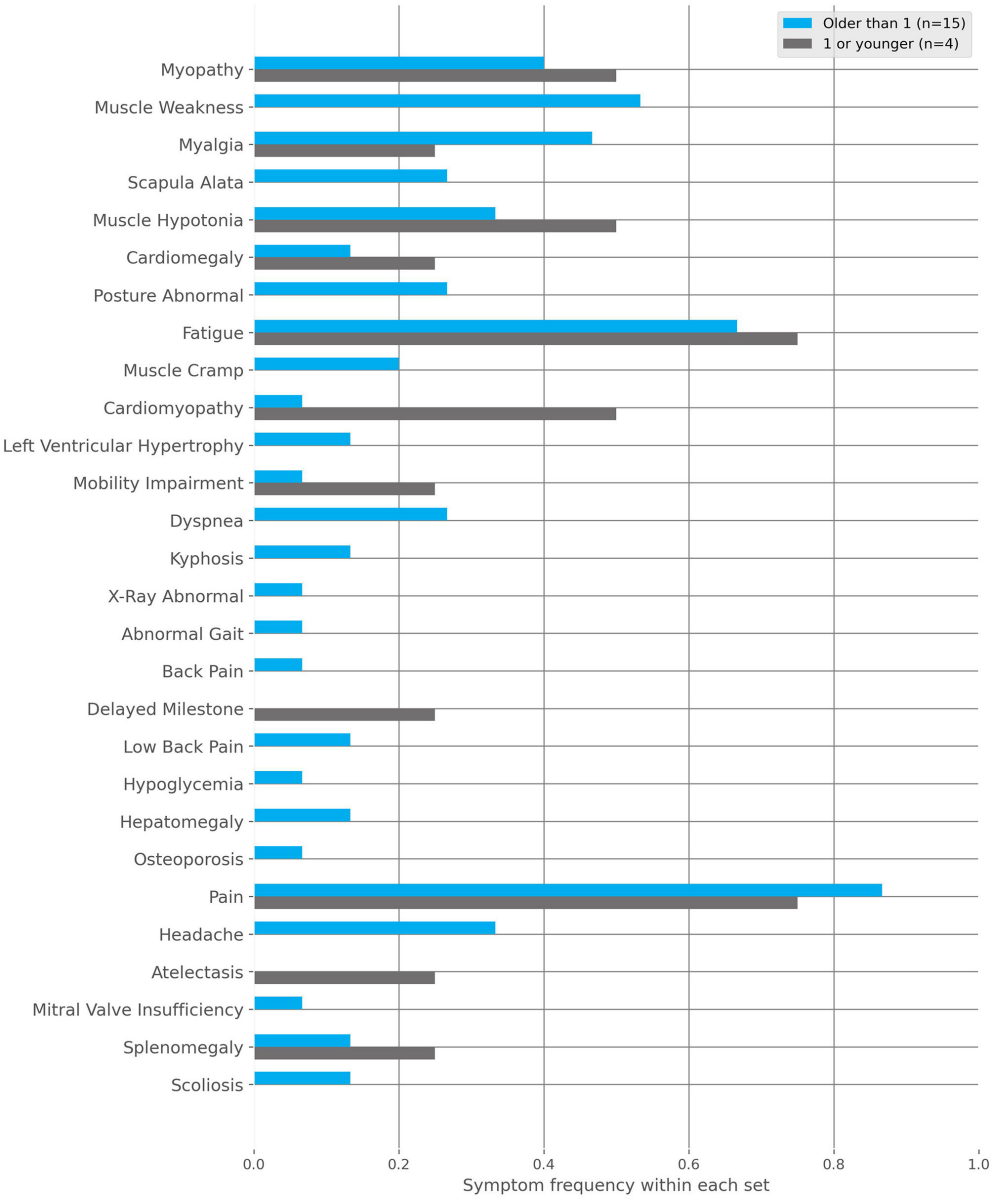
The frequencies of characteristics found within the Infantile-onset Pompe disease (IOPD) and late-onset Pompe disease (LOPD) subgroups to the positive cohort. These subgroups are defined by the first occurrence of symptoms, specifically before or after 1 year of age, respectively.

*IOPD*: Symptom onset at <1 year of age (Figure 5). Below are the most frequent characteristics experienced and the associated fraction of patients (*n* = 4):

- Muscle hypotony (frac = 0.50).
- Cardiomyopathy (frac = 0.50).
- Myopathy (frac = 0.25).
- Cardiomegaly (frac = 0.25).
- Pain (frac = 0.25).
- Atelectasis (frac = 0.25).
- Fatigue (frac = 0.25).
- Splenomegaly (frac = 0.25).
- Restricted mobility (frac = 0.25).

*LOPD*: Symptom onset at >1 year of age (Figure 5). Below are the fractions of patients (*n* = 15) where the following top 10 most frequent characteristics were documented:

- Pain (frac = 0.87).
- Fatigue (frac = 0.60).
- Myopathy (frac = 0.40).
- Muscle weakness (frac = 0.40).
- Myalgia (frac = 0.33).
- Headache (frac = 0.33).
- Scapula alata (frac = 0.27).
- Muscle hypotony (frac = 0.20).
- Dyspnea (frac = 0.20).
- Abnormal body posture (frac = 0.20).

## 4. Discussion

### 4.1. Screening efficiency

Currently, screening projects for Pompe disease are based on either manual review and consecutive dry blood spot test (DBS) or large-scale newborn DBS [also referred to as Newborn genetic screening (NBS)] to identify suspected patients for genetic testing. Governments have implemented fully funded and partly subsidized NBS in various regions worldwide (e.g., Japan, Taiwan, California-U.S.) (21–23). The advantages of NBS are that the sensitivity is expected to be close to 100% and the possibility of early intervention, which is critical for a better disease prognosis. Additionally, it is possible to prospectively follow up with patients where a high-risk of LOPD variant has been detected (38). However, one pitfall of this method is that Pompe disease patients that the healthcare system has missed in the past are never uncovered. Also, testing every newborn leads to very high false positive rates (79.55%−92.52%) and non-actionable information, which puts an unnecessary burden on parents and children, causing them psychological distress (21–23, 39–41). Additionally, genetic screening is challenged by continuously newly discovered genotype variants (42–48). For NBS, it usually requires manual screening via DBS of thousands of patients (5150 to 1895 newborns) to identify one single suspected patient for further genetic testing depending on the ethnicity and region (California, Japan, Taiwan) (21–23).

Our AI-based approach exploiting existing retrospective EHR data screened all patients (*n* = 350,116) admitted to any of the five hospitals of the SALK clinic group via a one time application (49). The AI operated at an efficiency of 5.47 (95% CI: 3.73–8.32) patients needed to be screened manually to identify one suspected patient. Five patients were identified as historically diagnosed patients in our dataset, which we considered must-not-miss patients, and all were identified by the AI and later confirmed by generalist physicians (five “diagnosed”) and specialist physicians (five “definite”) suggesting a low risk for false negatives.

Furthermore, frequently missed diagnoses of LOPD patients make the need for retrospectively identifying missed patients evident (9–13). Targeted manual approaches to screening patients before DBS and genetic testing have yielded respectable results (9–13). Table 4 shows an overview of the outcomes in other screening studies. However, this requires an additional clinical examination of patients to route them for further manual DBS screening, often following a multicenter approach over several years. On average, four eligible patients per month per clinic were found, while requiring 17 patients to be examined to find a single suspected patient (9–13, 50). Our AI-based screening enabled an analysis reaching back more than a decade (2007–2021). Inclusive of the review round with GPs and SPs, and including running the AI, the screening took <1 month and resulted in 21 eligible patients per clinic. From this cohort, we found it required 5.47 patients to be manually reviewed to find one suspected patient.

**Table 4:** Overview of published Pompe disease screening studies.

**Region**	**Duration in months**	**Number of clinics**	**Number of manually screened patients**	**Number of suspected patients**	**Number of confirmed patients**	**Study**
Austria	1.00	5.00	104.00	19.00	n/a	Current study
Italy	14.00	17.00	1,051.00	30.00	17.00	(12)
Spain	28.00	7.00	348.00	20.00	16.00	(10)
Turkey	12.00	4.00	98.00	6.00	3.00	(50)
Iran	12.00	1.00	93.00	5.00	3.00	(13)
Portugal	24.00	9.00	18.00	16.00	5.00	(9)
Germany, Great Britain	60.00	7.00	3,076.00	232.00	74.00	(11)
*Average of other studies*	*25.00*	*7.50*	*780.67*	*51.50*	*19.67*	
**Number needed to screen—suspected**	**Number needed to screen—confirmed**	**Discovered patients per month—overall**	**Discovered patients per month—clinic**	**Study**		
5.47	n/a	104.00	20.80	Current study		
35.03	1.76	75.07	4.42	(12)		
17.40	1.25	12.43	1.78	(10)		
16.33	2.00	8.17	2.04	(50)		
18.60	1.67	7.75	7.75	(13)		
1.13	3.20	0.75	0.08	(9)		
13.26	3.14	51.27	7.32	(11)		
*16.96*	*2.17*	*25.91*	*3.90*	*Average of other studies*		

The italic values indicate the arithmetic averages of the respective column calculated from all studies listed in this table other than the current study.

Due to the low prevalence of rare diseases, the number of patients screened is the most relevant variable for success. Thus, resource-efficient scalability and accuracy are key metrics for any prospective approach (39, 40). For NBS, as well as targeted manual screening, required time and resources increase almost directly proportional with the number of screened patients (20). Our AI-based approach scales with increasing resource efficiency for every additional patient included for screening. This indirect proportional behavior marks a distinct advantage for the screening of rare diseases.

The prevalence of Pompe disease patients in the greater Salzburg region, based upon our methodology is 1:18,427.16 (95% CI: 11,797.67–28,782.29), which falls into the same range as reported based upon genetic databases for (non-Finnish) Europeans (1:13,756) and global prevalence (1:23,232) (15).

### 4.2. Differentiating characteristics

We chose to use free-text documentation as the focus for the patient characteristic analysis as it not only showed the highest level of continuity but also reflects patients in early stages who did not have a laboratory workup yet. We considered this to be the most challenging part of the diagnostic patient journey. Analyzing the PCA, it appears that PC3 (Fatigue, Myalgia, and Dyspnea) drives a weak separation of the positive cohort from the negative and background groups. Notably, fatigue was found in all components to be an important feature. However, PC1 (Fatigue, Headache, and Lower Back Pain) includes more common symptoms as the other large contributors, while PC2 (Splenomegaly, Hepatomegaly, Fatigue) highlights features related to organs abnormalities. This suggests that symptoms related to muscular impairment are more specific and thus important when differentiating patients suspicious for suffering from Pompe disease. Scapula alata, myopathy, myalgia, muscle hypotony and muscle weakness were the characteristics pointing at Pompe disease with the highest statistic certainty among all patients identified by the model, which is in line with cardinal symptoms described in the literature (51–53). Although clinical courses can vary remarkably, especially in LOPD (54–56), this suggests that the AI identified patients correctly according to their phenotypes.

### 4.3. Phenotypical insights

Co-occurrence of cardiomegaly and cardiomyopathy being important differentiating characteristics (Figure 2), with muscle hypotony (Figure 4) shows agreement with IOPD differentiating characteristics described in the literature (18, 51). Interestingly, muscle hypotony does not co-occur more than expected with muscle weakness and myalgia. Of course, the limitation is the small sample size (*n* = 19); however, it might point at muscular tone being either less relevant when other neuromuscular characteristics are present or being more fulminant when others are missing (57). Further conversely to other general symptoms such as pain and headache, fatigue was not only frequently reported, but also coincides more than expected with muscle weakness and myalgia, suggesting a meaningful differentiating character. Similarly, we found that fatigue distinctly inversely coincided with cardiomegaly, normally found in IOPD, which suggests that certain symptoms might be present but are not reported, as in this case newborns do not report “fatigue” as such. Myalgia was an important differentiating characteristic but also the one together with muscle cramp which coincided with the most neuromuscular symptoms, which suggests that they serve as good predictors for Pompe disease when appearing in the context of neuromuscular diseases. Further Scapula alata was an important differentiating characteristic but also the one together with myopathy which was not found in the background population, which suggests that it serves as a good predictor for Pompe disease, when there are little other specific characteristics. Other frequently described characteristics such as dyspnea, back pain/lumbago and hepatomegaly (52, 53) were found in the positive group as well, however much more frequently in the negative group, which suggests that they are poor stand-alone predictive characteristics for identifying Pompe disease patients.

### 4.4. Established phenotypes

The phenotypes derived from the results of the AI are based upon EHRs provided by the SALK clinic group (see text footnote ^2^). The clinic group is servicing the area in and around Salzburg (Austria), including parts of Bavaria (Germany), Styria (Austria), and Upper Austria (Austria), thus representing the population in these regions (58).

Phenotype onset at <1 year of age (IOPD): In our cohort (*n* = 4), we have found cardiomyopathy (frac = 0.50) and muscle hypotony (frac = 0.50) to be the most common symptoms, which are also described as cardinal findings in IOPD patients (42, 43). Further organic abnormalities, such as cardiomegaly (frac = 0.25), splenomegaly (frac = 0.25) or atelectasis (frac = 0.25) as well as restricted mobility (frac = 0.25), but interestingly no scapula alata, had been documented. These results may indicate features that are less clinically prominent in newborns compared to older patients.

Phenotype onset at >1 year of age (LOPD): In our cohort (*n* = 15), no cardiac characteristic has been found under the top 10 most reported symptoms. However, characteristics related to proximal muscle weakness and progressive failing of the musculature have been found, which is in agreement with characterizations in the literature so far (16, 43, 59). In general, more unspecific symptoms like pain (frac = 0.87), fatigue (frac = 0.60) and headache (frac = 0.33), as well as a bigger spectrum of characteristics, point to the bigger variance of symptoms experienced in LOPD patients. This diversity is likely due to their more individual patient journeys (19, 51).

### 4.5. Limitations

A frequent limitation in rare disease studies are the small sample sizes, which challenge statistically significant findings. However, even so, we were still able to produce some statistically significant results, which is also due to our methodology, which enables the analysis of large datasets (350,116 EHRs) at once. In our methodology, we can only include patients who have at least visited secondary care. However, while this will bias the sampling, this is usually also where Pompe disease patients are identified, worked up and, hopefully, diagnosed. Furthermore, our AI can only assess what is documented, i.e., present in the EHRs. Compared to survey-based investigations, patients are not interviewed directly, and their subjective impressions are not recorded. However, while this will result in some loss of individual data granularity, retrospective data is readily available and does not suffer from recall nor memory bias.

Another potential limitation is that the data extraction for analysis was automated, which can result in some data loss or “machine” bias compared to the manual curation of data sets. However, at the same time, this standardized approach prevents the introduction of multiple biases, as multiple human agents curate data burdened by subjectivity. Also, this shows the potential for a scalable data analysis framework, which is a core requirement in any feasible solution to the rare disease conundrum. Furthermore, descriptive statistics and manual quality checks can mitigate inherent biases.

One limitation of this study remains the risk of producing false negatives. Due to the nature of a large-scale screening test, the number of “non-flagged” patients is overwhelming and a systematic manual review not feasible. However, the *in-silico* performance as well as prevalence based upon the results of the AI, which largely agrees with numbers reported in the scientific literature, suggest that a systematic error leading to potential false negatives is at least not obvious (14, 15).

Further, the AI was able to find all historically diagnosed patients, which suggests a highly sensitive screening. Additionally, to mitigate a subjective bias, the assessments were done in two iterative rounds. Firstly, with a team of well-instructed generalist physicians and then together with feedback from key opinion leaders for Pompe disease. There is a clear value in demonstrating the robustness of the AI as a screening tool by showing that “flagged” patients have medical histories that disease specialists agree with.

Lastly, the review of EHRs to validate the quality of suspicion of flagged patients was performed on anonymized data. The clinical workup is not part of this retrospective data analysis study, which leaves a gap in the final confirmation of Pompe disease patients. However, this study design was consciously chosen as to honor the four ethical principles of the ethics guidelines for Trustworthy AI^3^ released by the EU: “respect for human autonomy,” “prevention of harm,” “fairness,” “explicability.” Thus, only anonymous records were taken into account for this study, and a de-anonymization would be beyond the scope of this retrospective feasibility study. However, an agreement of disease specialists with the results provided by the AI, distinctly demonstrates its potential capacity as a rare disease screening tool.

### 4.6. Outlook

Our proposed approach is an automated process optimized to run in the background, thoroughly screening every single patient visiting the respective healthcare facility. The found screening efficiency, as well as phenotypical insights, are compelling outcomes for this methodology. Our AI-based approach inserts itself into the existing landscape of screening approaches by markedly improving upon targeted manual screenings. It perfectly complements NBS while showing its potential as a stand-alone resource-efficient alternative. In addition, the possibility for automated analysis of retrospective EHRs opens up a whole spectrum of different possibilities ranging from predictive analytics to deep phenotyping for precision medicine. Further investigations for also other rare and complex diseases, as well as the inclusion of a prospective clinical component, will be useful to reveal and proof the full potential of this approach.

## 5. Conclusion

This study shows how an AI-based approach analyzing retrospective EHRs results in resource-efficient identification and automated phenotyping of Pompe disease patients. Using this approach, we discovered novel insights into differentiating characteristics of suspected Pompe disease patients. We were further able to approximate the prevalence for Pompe disease for the region covered by the EHRs. Lastly, we showed the feasibility of implementing this approach into existing hospital workflows. Our results demonstrate the potential of a scalable solution enabling systematic identification of rare disease patients and phenotypes. Therefore, this methodology can potentially improve both the timing and accuracy of identifying rare disease patients. In this study, we highlight Pompe disease, a rare, progressively debilitating, but treatable neuromuscular disease. However, implementing this methodology for all rare diseases should be encouraged to ultimately lead to better care for all patients.

## Data availability statement

The data analyzed in this study is subject to the following licenses/restrictions. The datasets analyzed during the current project are not publicly available due to legal agreements made with the providing institution. Aggregated data in the form of tables are available from the corresponding author on reasonable request and subject to institutional approval. Requests to access these datasets should be directed at: science@symptoma.com.

## Author contributions

SL, JN, EA, and AM contributed to the conception and design of the study. SL, RW-O, SG, HM, VP, FL, and EA compiled the database and implemented the methodology. SL, HM, and AM performed the data analysis. SL wrote the first manuscript draft. SL, JN, and AM revised the manuscript critically. All authors contributed to the article and approved the submitted version.

## Footnotes

^1^ http://www.symptoma.com

^2^Landeskliniken Salzburg (SALK) https://salk.at/.

^3^ https://digital-strategy.ec.europa.eu/en/library/ethics-guidelines-trustworthy-ai

## Supplementary material

Supplementary Material S1 Results of the benchmarking study of Symptoma's AI for Pompe disease.
