# Supplementary material for: An artificial intelligence-based approach for identifying rare disease patients using retrospective electronic health records applied for Pompe disease
Source: Front Neurol. 2023 Apr 21;14:1108222. doi: 10.3389/fneur.2023.1108222 (PMC10160659; doi:10.3389/fneur.2023.1108222)
Supplement: Supplementary Material S1 — Results of the benchmarking study of Symptoma's AI for Pompe disease. [file Data_Sheet_1.DOCX]

# Supplementary Material S1

In order to benchmark the performance of the AI we have analyzed artificial cases built from symptom frequencies of Pompe disease patients found in the scientific literature^[[1]](#footnote-1)^. We have applied the strictest threshold for the symptom frequencies by using the lower end of the described symptom frequency brackets.^[[2]](#footnote-2)^ The control cases are based on the best practice case report library of BMJ^[[3]](#footnote-3)^, which show at least one symptom that overlaps with the clinical presentation of Pompe disease. The methodology for this analysis has been described in a previously published study^[[4]](#footnote-4)^.

The results are the following calculated with 95% confidence intervals [CI]:

- F1 score when considering top-1 causes as predicted positive: 0.967, 95% CI [0.925, 1.0]
- F1 score when considering top-3 causes as predicted positive: 0.973, 95% CI [0.936, 1.0]
- F1 score when considering top-5 causes as predicted positive: 0.98, 95% CI [0.947, 1.0]
- F1 score when considering top-10 causes as predicted positive: 0.983, 95% CI [0.947, 1.0]
- F1 score when considering top-30 causes as predicted positive: 0.981, 95% CI [0.940, 1.0]
- MRR: 0.947, 95% CI [0.884, 1.0]
- ROC-AUC: 0.987, 95% CI [0.962, 1.0]


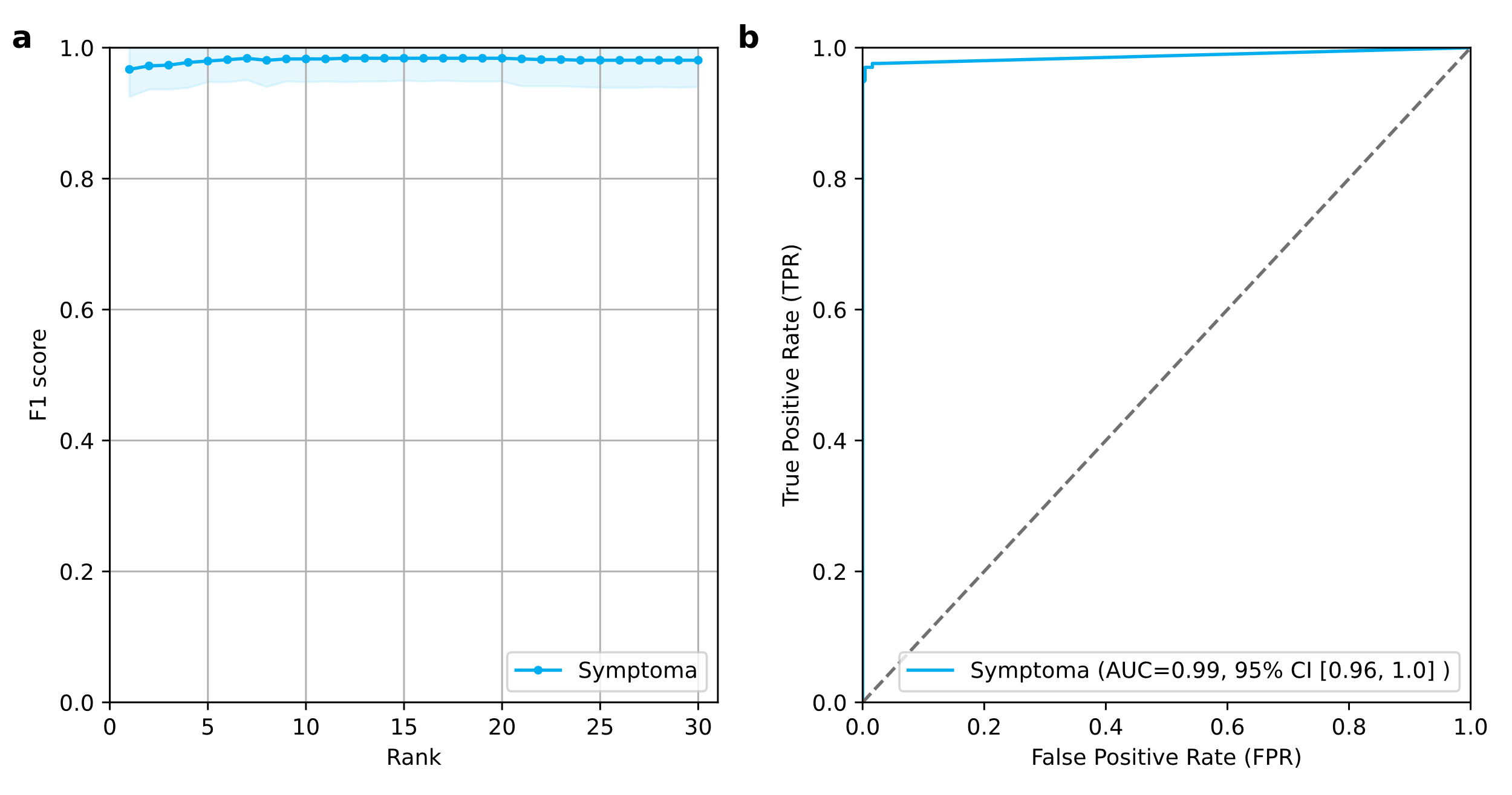
**Figure 1.** Performance of Symptoma in identifying Pompe disease cases. On the left (a), we detail the F1 score of Symptoma for each rank. On the right (b), we show the Receiver Operating Characteristic (ROC) curve. The area under the curve (AUC) with 95% confidence intervals (CI) is provided to summarize the curve. The blue band shows the 95% CI.

1. <https://www.orpha.net/consor/cgi-bin/Disease_HPOTerms.php?lng=DE&data_id=14&Disease_Disease_HPOTerms_diseaseGroup=Pompe-Krankheit&Disease_Disease_HPOTerms_diseaseType=Pat&Krankheite(n)/Krankheitsgruppe=Glykogenose-Typ-2&title=Glykogenose%20Typ%202&search=Disease_HPOTerms_Simple> (Accessed 13^th^ of February 2023) [↑](#footnote-ref-1)
2. <https://www.orphadata.com/phenotypes/> (Accessed 13^th^ of February 2023) [↑](#footnote-ref-2)
3. <https://bestpractice.bmj.com/info/> (Accessed 13^th^ of February 2023) [↑](#footnote-ref-3)
4. Munsch N, Martin A, Gruarin S, Nateqi J, Abdarahmane I, Weingartner-Ortner R, Knapp B. Diagnostic Accuracy of Web-Based COVID-19 Symptom Checkers: Comparison Study. J Med Internet Res. 2020 Oct 6;22(10):e21299. doi: 10.2196/21299. PMID: 33001828; PMCID: PMC7541039. [↑](#footnote-ref-4)
